# Association between dietary selenium and zinc intake and risk of dilated cardiomyopathy in children: a case-control study

**DOI:** 10.1186/s12887-024-04706-1

**Published:** 2024-04-11

**Authors:** Maryam Aryafar, Mohammad Mahdavi, Hossein Shahzadi, Yeganeh Rajabpour Ranjbar, Mohammad Hassan Sohouli, Sina Afzal, Asal Neshatbini Tehrani, Danial Fotros, Ghazal Daftari

**Affiliations:** 1 Rajaei Cardiovascular Medical and Research Center, Iran University of Medical Sciences Tehran, Tehran, Iran; 2 Department of Clinical Nutrition and Dietetics, Faculty of Nutrition Sciences and Food Technology, Research Institute, National Nutrition and Food Technology, Shahid Beheshti University of Medical Sciences, Tehran, Iran; 3 Student Research Committee, Shahid Beheshti University of Medical Sciences, Tehran, Iran; 4 Department of Orthopedic and Trauma Surgery, Shahid Beheshti University of Medical Sciences, Tehran, Iran; 5 Student Research Committee, Ahvaz Jundishapur University of Medical Sciences, Ahvaz, Iran; 6 Department of Nutrition, School of Allied Medical Sciences, Ahvaz Jundishapur University of Medical Sciences, Ahvaz, Iran; 7 School of Medicine, Tehran University of Medical Sciences, Tehran, Iran; 8 Keshavarz Boulevard, Tehran, Iran

**Keywords:** Dilated cardiomyopathy, Selenium, Zinc, Pediatrics, Case-control study

## Abstract

**Background:**

Dilated cardiomyopathy (DCMP) is characterized by the enlargement and weakening of the heart and is a major cause of heart failure in children. Infection and nutritional deficiencies are culprits for DCMP. Zinc is an important nutrient for human health due to its anti-oxidant effect that protects cell against oxidative damage. This case-control study aimed to investigate the relationship between dietary intake of zinc and selenium and the risk of DCMP in pediatric patients.

**Methods:**

A total of 36 DCMP patients and 72 matched controls were recruited, and their dietary intakes were assessed via a validated food frequency questionnaire. We used chi-square and sample T-test for qualitative and quantitative variables, respectively. Logistic regression analysis was applied to assess the relationship between selenium and zinc intake with the risk of DCMP.

**Results:**

After fully adjusting for confounding factors, analyses showed that selenium (OR = 0.19, CI = 0.057–0.069, P trend < 0.011) and zinc (OR = 0.12, CI = 0.035–0.046, P trend < 0.002) intake were strongly associated with 81% and 88% lower risk of pediatric DCMP, respectively.

**Conclusions:**

This study highlights the protective role of adequate dietary intake of selenium and zinc in decreasing the risk of DCMP in children. Malnutrition may exacerbate the condition and addressing these micronutrient deficiencies may improve the cardiac function. Further studies are recommended to detect the underlying mechanisms and dietary recommendations for DCMP prevention.

## Introduction

Dilated cardiomyopathy (DCMP) is a condition characterized by systolic dysfunction and biventricular or left ventricle dilation in the lack of predisposing factors such as coronary artery disease, hypertension or valvular disorders which cause systolic dysfunction [1]. DCMP usually begins in the left ventricle by thinning and stretching of the heart muscles resulting in enlargement of heart chambers that impairs the normal contraction and blood pumping [2]. DCMP presents with orthopnea, heart failure, breathlessness, impaired exercise tolerance, poor feeding, fatigue and sweating [3]. The prevalence of DCMP is estimated from 1:500 to 1:2500 in general population [4, 5]. In children, the incidence rate is about 0.57 per 100,000 population annually and is higher in boys than girls [6]. In children, the common causes of DCMP is idiopathic, neuromuscular disorders such as Becker and Duchen dystrophies, nutritional deficiencies and inflammation [7]. Paediatric DCMP is observed to occur after influenza, parvo virus B19, coxsackie, herpes, Ebstein Barr, adeno and human immunodeficiency viruses [7]. Furthermore, taurine, calcium, zinc and selenium depletion decrease the heart contractility and are implicated in DCMP [8].

Selenium and zinc, micronutrients found in red meat, grains, nuts and sea foods is an indispensable elements in human health [9]. Selenium in the form of selenocysteine and as the 21st amino acid is incorporated in the selenoproteins, modulating immunologic, cardiovascular and metabolic functions via anti-inflammatory, anti-cancer and anti-oxidant effects [10]. Also, selenium is crucial for sperm motility and thyroid function [11]. Gluthatione peroxidases, iodothyronine deiodinases and thioredoxin reductases are anti-oxidant enzymes depending on selenium [12, 13]. Zinc is an important element in apoptosis and cellular membrane stability [14]. In addition, it is a necessary component of enzymes such as angiotensinogen converting enzyme and carbonic anhydrases that are regulators of acid-base balance and fluid homeostasis [15]. Zinc is a co-factor of different enzymes that contribute to function of anti-oxidant systems. It stabilizes cellular membrane and protects the cells against oxidative conditions. In addition, zinc inhibits pro-oxidant enzymes and decreases reactive oxygen species production in stress conditions [16–18].

Several studies have demonstrated that selenium deficiency may contribute to cardiovascular disorders such as Keshan disease, which is an endemic DCMP in China [19]. It is also indicated that zinc deficiency is culprit of cardiac cellular damage and decreased cardiac function [20, 21]. It is suggested that high selenium and zinc intake may reduce the risk of cardiovascular incidence and mortality [22]. Considering previous studies, little is known about the selenium and zinc intake in children with DCMP, thus we aimed to explore the association of dietary intake of selenium and zinc with DCMP risk in children.

## Methods and materials

### Study population

In this case-control study, 45 patients within the age range of 2–17 years old who had been diagnosed with idiopathic DCMP for at least 6 months, were recruited from Rajaie cardiovascular, medical and research center during spring and summer of 2022 in Tehran, Iran. Physical exam, electrocardiogram (ECG), clinical history, echocardiography and chest X-ray had been used for diagnosis.

Inclusion criteria were individuals with signs and symptoms of heart failure such as low exercise tolerance, fatigue, edema and shortness of breath. Exclusion criteria were following: having renal failure, diabetes mellitus, malignancies, infectious disease, pregnancy, valvular, rheumatic, hypertensive and congenital heart diseases and also, life expectancy less than 6 months. Among 45 patients that were initially identified, 9 patients were excluded due to the high risk of mortality. Also, 72 controls were matched according to sex and age. The controls were randomly allocated patients admitted to other wards of the same hospital with no history of cardiovascular diseases, confirmed with echocardiography. It is important to mention that when the cases are selected from hospital, controls from hospital are preferred over community-based control selection. The protocol of this study was approved by Rajaie cardiovascular, medical and research center ethics committee (IR.RHC.REC.1401.016). All the parents / legal guardian of participants were informed about the study and signed the written informed consent form.

### Dietary intake assessment

A reproducible and valid food frequency questionnaire (FFQ) [23–25] was used to collect dietary intake. A trained dietician collected the portion size and frequency of food items based on daily, weekly, monthly or yearly intake. The dietary intakes of participants were checked with their parents to reduce the recall bias. We used Nutritionist IV [26] to analyse the collected data and The United States Department of Agriculture (USDA) Food Composition Table (FCT) to calculate nutrients and energy contents.

### Data collection

Socio-demographic and anthropometric information of the participants were collected by a trained interviewer. Body weight was measured to the nearest 100 g while standing on digital scales (Soehnle, Berlin, Germany). Height was calculated by a non-stretch portable meter to the nearest 0.5 cm. Body mass index (BMI) was measured by dividing of weight in kilograms to square of height in meter.

### Statistical analysis

After assessing the normality of the variable’s distribution by Kolmogorov-Smirnov test, independent sample T-test was used to compare quantitative variables between the two groups, as Chi-square was also used for qualitative variables. The baseline characteristics were reported as mean ± standard deviation (SD) for quantitative variables, and number for qualitative variables. The association of selenium/zinc with the odds of cardiomyopathy was assessed by applying logistic regression. The analyses were adjusted for probable confounders, including age, sex, BMI, energy, fiber, Na and K. All analyses were performed using statistical package software for social science (SPSS) 22.0 statistical software, and *P*-value less than 0.05 was considered statistically significant.

## Results

### Baseline characteristics

The mean ± SD for the age across case and control groups were 9.83 ± 4.55 and 8.7 ± 1.54 years, respectively. There was a significant difference in the distribution of sex between cases and controls (*p* < 0.001), with a higher proportion of males in the control group. However, no significant differences were observed in weight (*p* = 0.208) and BMI (*p* = 0.702) between the two groups (Table 1).

**Table 1:** Baseline General Characteristics of Study Participants

	Case (*n* = 36)	Control (*n* = 72)	*P* value^1^
Sex (n)^1^ Male Female	36 15(41.6%) 21(58.4)	72 58(80.5) 14(19.5)	< 0.001
Age (y)^2^	9.83 ± 4.55	8.7 ± 1.54	0.157
Weight (kg)^2^	35.38 ± 21.96	30.49 ± 9.47	0.208
BMI (kg/m^2^)^2^	16.9 ± 4.8	17.25 ± 3.58	0.702

Data are presented as frequency, percent and mean ± standard deviation

^1^ Pearson chi-square test

^2^ Independent t-test

### Dietary intakes

The dietary intakes of study participants across the case and control groups are presented in Table 2. Significant differences were found in the intake of protein, carbohydrate, total fiber, fat, cholesterol, sodium, potassium, vitamin A.RE, vitamin C, iron, vitamin D, vitamin E, folate, B12, magnesium, zinc, and selenium between cases and controls (*p* < 0.05 for all). Cases generally exhibited lower intakes of these nutrients compared to controls.

**Table 2:** Dietary intakes of Study Participants

	Case (*n* = 36)	Control (*n* = 72)	*P* value
Protein (gr)	74.72 ± 34.43	115.22 ± 33.5	< 0.001
Carbohydrate (gr)	247.56 ± 88.89	569.94 ± 227.55	< 0.001
Total fiber (gr)	34.36 ± 18.41	8.4 ± 4.55	< 0.001
Fat (gr)	74.28 ± 30.6	115.48 ± 53.29	< 0.001
Cholesterol (mg)	264.86 ± 107.93	348.69 ± 145.98	0.003
SFA (gr)	23.23 ± 9.21	19.37 ± 34.19	0.372
Sodium (mg)	3836.71 ± 1624.36	5466.51 ± 3986.41	0.003
Potassium (mg)	3526.01 ± 1599.71	8577.74 ± 3938.85	< 0.001
Vitamin A.RE (mcg)	604.63 ± 362.26	3173.66 ± 2186.49	< 0.001
Vitamin C (mg)	169.23 ± 114.82	409.45 ± 369.28	< 0.001
Calcium (mg)	1294.89 ± 576.89	1342.24 ± 409.84	0.662
Iron (mg)	15.65 ± 9.44	47.39 ± 47.97	< 0.001
Vitamin D (mcg)	2.59 ± 1.71	16.21 ± 24.74	< 0.001
Vitamin E (mg)	11.23 ± 5.03	15.83 ± 14.25	0.016
Folate (mcg)	434.73 ± 181.89	621.89 ± 285.73	< 0.001
B12 (mcg)	5.51 ± 3.33	9.39 ± 10.24	0.004
Magnesium (mg)	316.09 ± 134.29	531.45 ± 211.95	< 0.001
Zinc (mg)	10.27 ± 4.76	13.44 ± 4.67	0.002
Selenium (mcg)	0.09 ± 0.04	0.13 ± 0.05	< 0.001

Independent t-test

Data are presented as mean ± standard deviation

### Odds ratios for cardiomyopathy

The odds ratio (ORs) and 95% confidence interval (CIs) for the occurrence of cardiomyopathy based on the tertiles of selenium and zinc intake are reported in Table 3. In the crude model, selenium and zinc were inversely associated with the lower odds of cardiomyopathy, with an OR of 0.144 and 0.179 for the highest tertile, respectively (*P* < 0.05 for trend). Furthermore, after adjusting for age, sex, BMI, energy, fiber, Na and K (in the fully adjusted model), in the highest versus lowest tertile of selenium and zinc, the lower odds of cardiomyopathy remained significant (OR = 0.198, 95% CI: 0.05–0.69; *P* value = 0.011 for trend and OR = 0.127, 95% CI: 0.03–0.46; *P* value = 0.002 for trend, respectively).

**Table 3:** Odds and 95% confidence interval for occurrence of cardiomyopathy in each tertile of selenium and zinc intake

Tertiles of selenium intake				
	T1	T2	T3	*P* value
**Selenium**				
Model 1	1 (ref)	0.447 (0.172, 1.160)	0.144 (0.046, 0.455)	0.001
Model 2	1 (ref)	0.523 (0.185, 1.481)	0.180 (0.053, 0.611)	0.006
Model 3	1 (ref)	0.433 (0.137, 1.376)	0.198 (0.057, 0.693)	0.011
**Zinc**				
Model 1	1 (ref)	0.394 (0.150, 1.033)	0.179 (0.060, 0.534)	0.002
Model 2	1 (ref)	0.331 (0.111, 0.984\5)	0.134 (0.039, 0.465)	0.001
Model 3	1 (ref)	0.342 (0.108, 1.086)	0.127 (0.035, 0.466)	0.002

Model 1: Crude

Model 2: Adjustment for age, sex

Model 3: Additional adjustment for BMI, energy, fiber, Na, K, FBS, CRP

## Discussion

The present case-control study demonstrated that lower intake of macronutrients and micronutrients such as selenium and zinc is associated with higher risk of DCMP in children, after fully adjusting of confounding factors such as energy, BMI, age, sex, fiber, Na and K.

In our study, dietary intake of macronutrients was significantly lower in cases than controls. Align with our finding Ocal et al. reported that children with malnutrition exhibit cardiovascular disorder including arrythmia, sudden death, heart failure and dilated cardiomyopathy [27]. Also, gross examination of the myocardium in malnourished children showed a flabby, pale and thin-walled heart [28]. Nutritional interventions may improve the quality of life and myocardial function. Failure to thrive is one of the most important problems in children with cardiomyopathy. Adequate intake of macronutrients may improve cardiac function and also, specific micronutrients decrease the myocardial abnormalities that occur in cardiomyopathies and heart failure [29].

There is a vicious circle between malnutrition and DCMP. Poor nutritional status is a cause of DCMP and on the other hand, DCMP leads to malnutrition through metabolic disturbances, chronic inflammation and gastrointestinal malabsorption [30–32]. The inflammatory condition in most chronic diseases such as DCMP affects the metabolism and results in reduced cardiac muscle function and mass over time [33]. Different micronutrients deficiency may cause DCMP. Vema et al. reported a 15-month-old child with DCMP caused by hypocalcemia nutritional rickets that responded to vitamin D and calcium supplementation and systolic function normalized after 3 months [34].

Consistent with our study, Ripa et al. reported that primary or secondary zinc deficiency may result in DCMP and also, reduced plasma level of zinc is an important prognostic and diagnostic marker for DCMP [35]. In addition, Topuzoglu et al. demonstrated that patients with DCMP have lower plasma level of zinc compared with healthy controls [36]. Zinc is an important component of various enzymes such as superoxide dismutase. An impairment in superoxide dismutase function leads to reaction of superoxide anions with hydrogen peroxide and production of hydroxide radicals that induce cell damage. Zinc protects the cells against free radicals and thus decreases the cardiovascular disorders. In DCMP and consequently heart failure, activation of atrial natriuretic peptide (ANP) causes high urinary excretion of zinc, concluding to zinc deficiency and impaired cardiac performance [37]. On the other hand, Chou et al. did not find any association between patients with DCMP and control group [38]. The differences in reports may be due to sample size, dietary food intake and methodology.

Another micronutrient that is protective in cardiovascular diseases is selenium. Dasgupta et al. reported a 14 years old boy with severe malnutrition, selenium deficiency and heart failure that has been treated with selenium replacement and nutritional support and become completely asymptomatic after four weeks [33]. In line with our study, Khater et al. demonstrated that pediatric patients with DCMP have reduced plasma level of selenium and this element can prevent myocardial damage [39]. Frustaci et al. indicated that in patients with intestinal malabsorption, a reversible selenium and zinc DCMP may occur oxidative damages to cell membrane, increased cell autophagy and decreased anti-oxidant activity [40]. Furthermore, Basil et al. reported that selenium level is significantly lower in patients with DCMP in comparison with control group [41]. In contrast to our study, Cunha et al. investigated that there is no difference between selenium level in patients with DCMP and control group [42]. It may be due to different food pattern and sample size. Selenium is a crucial element in inflammation and immunity and improves antioxidant reserve and suppresses production of tumor necrosis factor alfa and interleukins. Selenium deficiency may also play a role in myocardial damage and recovery.

Current study has some strengths including the analysis of various macro and micronutrients and also adjusting the confounding factors to improve the reliability of the study. There are some limitations, due to case-control nature of the study we could not establish a causative relationship between selenium and zinc intake with DCMP and the possibility of recall bias is another issue to consider.

## Conclusion

In conclusion, current study concluded that there is an inverse association between macronutrients, selenium and zinc intake with the risk of pediatric DCMP. Further studies are needed to evaluate the amount of selenium and zinc intake to prevent DCMP.

## Data Availability

The datasets examined in the current study are available from the corresponding author on. reasonable request.

## Abbreviations

DCMP: dilated cardiomyopathy
ECG: electrocardiogram
FFQ: food frequency questionnaire
USDA: the United States department of agriculture
FCT: food composition table
BMI: body mass index
SD: standard deviation
SPSS: statistical package software for social science
OR: odds ratio
CI: confidence interval
SFA: saturated fatty acid
RE: retinol equivalent

## References

[1] Elliott P, Andersson B, Arbustini E, Bilinska Z, Cecchi F, Charron P (2008). Classification of the cardiomyopathies: a position statement from the European Society of Cardiology Working Group on Myocardial and Pericardial diseases. Eur Heart J.

[2] Ampong I (2022). Metabolic and metabolomics insights into dilated cardiomyopathy. Annals Nutr Metabolism.

[3] Bailer J, Kaufman BD (2010). Nutrition implications of heart failure and heart transplantation in children with dilated cardiomyopathy: a case series. ICAN: Infant Child Adolesc Nutr.

[4] Weintraub RG, Semsarian C, Macdonald P (2017). Dilated cardiomyopathy. Lancet.

[5] Reichart D, Magnussen C, Zeller T, Blankenberg S (2019). Dilated cardiomyopathy: from epidemiologic to genetic phenotypes: a translational review of current literature. J Intern Med.

[6] Towbin JA, Lowe AM, Colan SD, Sleeper LA, Orav EJ, Clunie S (2006). Incidence, causes, and outcomes of dilated cardiomyopathy in children. JAMA.

[7] Amrita M, Amar T. Dilated cardiomyopathy in children: early detection and treatment. Cureus. 2022;14(11). 10.7759/cureus.31111 PMC9720092 36475220

[8] Carvajal K, Moreno-Sánchez R (2003). Heart metabolic disturbances in cardiovascular diseases. Arch Med Res.

[9] Rayman MP (2012). Selenium and human health. Lancet.

[10] Shan H, Yan R, Diao J, Lin L, Wang S, Zhang M (2015). Involvement of caspases and their upstream regulators in myocardial apoptosis in a rat model of selenium deficiency-induced dilated cardiomyopathy. J Trace Elem Med Biol.

[11] Rayman MP (2000). The importance of selenium to human health. Lancet.

[12] Lu J, Holmgren A, Selenoproteins (2009). J Biol Chem.

[13] Reeves M, Hoffmann P (2009). The human selenoproteome: recent insights into functions and regulation. Cell Mol Life Sci.

[14] Wood R, Ronnenberg A, King J, Cousins R, Dunns J, Burk R, Levander O. Modern nutrition in health and disease. Lippincott’s Illustrated Reviews Biochemistry, Lippincott Williams & Wilkins; 2005. pp. 248–70.

[15] Cohen N, Golik A (2006). Zinc balance and medications commonly used in the management of heart failure. Heart Fail Rev.

[16] Marreiro DDN, Cruz KJC, Morais JBS, Beserra JB, Severo JS, De Oliveira ARS (2017). Zinc and oxidative stress: current mechanisms. Antioxidants.

[17] Ruz M, Carrasco F, Rojas P, Codoceo J, Inostroza J, Basfi-Fer K (2013). Zinc as a potential coadjuvant in therapy for type 2 diabetes. FoodNutr Bull.

[18] Chasapis CT, Loutsidou AC, Spiliopoulou CA, Stefanidou ME (2012). Zinc and human health: an update. Arch Toxicol.

[19] Loscalzo J (2014). Keshan disease, selenium deficiency, and the selenoproteome. N Engl J Med.

[20] McKeag NA, McKinley MC, Woodside JV, Harbinson MT, McKeown PP (2012). The role of micronutrients in heart failure. J Acad Nutr Dietetics.

[21] Prasad AS. Zinc deficiency: has been known of for 40 years but ignored by global health organisations. British Medical Journal Publishing Group; 2003. pp. 409–10.

[22] Kuria A, Tian H, Li M, Wang Y, Aaseth JO, Zang J, Cao Y (2021). Selenium status in the body and cardiovascular disease: a systematic review and meta-analysis. Crit Rev Food Sci Nutr.

[23] Asghari G, Rezazadeh A, Hosseini-Esfahani F, Mehrabi Y, Mirmiran P, Azizi F (2012). Reliability, comparative validity and stability of dietary patterns derived from an FFQ in the Tehran lipid and glucose study. Br J Nutr.

[24] Azizi F, Hedayati M, Hosseini Esfahani F, Mehrabi Y, Mirmiran P (2010). Reliability and relative validity of an FFQ for nutrients in the Tehran lipid and glucose study. Public Health Nutr.

[25] Rezazadeh A, Omidvar N, Tucker KL (2020). Food frequency questionnaires developed and validated in Iran: a systematic review. Epidemiol Health.

[26] Hearst C, First IVD, Nutritionist. IV: diet analysis. San Bruno, CA: First DataBank. 1995.

[27] Öcal B, Ünal S, Zorlu P, Tezic H, Oğuz D (2001). Echocardiographic evaluation of cardiac functions and left ventricular mass in children with malnutrition. J Paediatr Child Health.

[28] Garson A. The science and practice of pediatric cardiology. (No Title). 1998.

[29] Miller TL, Neri D, Extein J, Somarriba G, Strickman-Stein N (2007). Nutrition in pediatric cardiomyopathy. Prog Pediatr Cardiol.

[30] Rahman A, Jafry S, Jeejeebhoy K, Nagpal AD, Pisani B, Agarwala R (2016). Malnutrition and cachexia in heart failure. J Parenter Enter Nutr.

[31] Sciatti E, Lombardi C, Ravera A, Vizzardi E, Bonadei I, Carubelli V (2016). Nutritional deficiency in patients with heart failure. Nutrients.

[32] Marinescu V, McCullough PA (2011). Nutritional and micronutrient determinants of idiopathic dilated cardiomyopathy: diagnostic and therapeutic implications. Expert Rev Cardiovasc Ther.

[33] Dasgupta S, Aly AM. Dilated cardiomyopathy induced by chronic starvation and selenium deficiency. Case Reports in Pediatrics. 2016;2016. 10.1155/2016/8305895 PMC5138453 27994905

[34] Verma S, Khadwal A, Chopra K, Rohit M, Singhi S (2011). Hypocalcemia nutritional rickets: a curable cause of dilated cardiomyopathy. J Trop Pediatr.

[35] Ripa S, Ripa R, Giustiniani S (1998). Are failured cardiomyopathies a zinc-deficit related disease? A study on Zn and Cu in patients with chronic failured dilated and hypertrophic cardiomyopathies. Minerva Med.

[36] Topuzoglu G, Erbay AR, Karul AB, Yensel N (2003). Concentations of copper, zinc, and magnesium in sera from patients with idiopathic dilated cardiomyopathy. Biol Trace Elem Res.

[37] Nouraei SM, Ghayemian A, Salehifar E (2007). Serum zinc level in dilated and ischemic cardiomyopathy. J Tehran Univ Heart Cent.

[38] Chou H, Yang H, Tsou S, Ho R, Pai P, Hsu H (1998). Status of trace elements in patients with idiopathic dilated cardiomyopathy in central Taiwan. Zhonghua Yi Xue Za Zhi = Chinese Medical Journal. Free China ed.

[39] Abdellatif GM, Morsy SM, Khater NM, Ahmed HS (2020). The role of Selenium Deficiency in dilated cardiomyopathy. Zagazig Univ Med J.

[40] Frustaci A, Sabbioni E, Fortaner S, Farina M, del Torchio R, Tafani M (2012). Selenium-and zinc‐deficient cardiomyopathy in human intestinal malabsorption: preliminary results of selenium/zinc infusion. Eur J Heart Fail.

[41] Basil OMS, Lewai SAA, Thair IAB. Selenium status and echocardiographic parameters in Iraqi patients with idiopathic dilated cardiomyopathy. 2007.

[42] Cunha Sd A, Filho FM, Bastos VLFC, Antelo DS (2002). Souza MMd. Thiamin, selenium, and copper levels in patients with idiopathic dilated cardiomyopathy taking diuretics. Arquivos brasileiros de cardiologia.

